# Comparison of *LiST* measles mortality model and WHO/IVB measles model

**DOI:** 10.1186/1471-2458-11-S3-S33

**Published:** 2011-04-13

**Authors:** Wei-Ju Chen

**Affiliations:** 1 Department of International Health, Johns Hopkins Bloomberg School of Public Health, Baltimore, Maryland, USA

## Abstract

**Background:**

The Lives Saved Tool (*LiST*) has been developed to estimate the impact of health interventions and can consider multiple interventions simultaneously. Given its increasing usage by donor organizations and national program planner, we compare the *LiST* measles model to the widely used World Health Organization's Department of Immunization, Vaccines and Biologicals (WHO/IVB) measles model which is used to produce estimates serving as a major indicator of monitoring country measles epidemics and the progress of measles control.

**Methods:**

We analyzed the WHO/IVB models and the *LiST* measles model and identified components and assumptions held in each model. We contrasted the important components, and compared results from the two models by applying historical measles containing vaccine (MCV) coverages and the default values of all parameters set in the models. We also conducted analyses following a hypothetical scenario to understand how both models performed when the proportion of population protected by MCV declined to zero percent in short time period.

**Results:**

The WHO/IVB measles model and the *LiST* measles model structures differ: the former is a mixed model which applies surveillance data adjusted for reporting completeness for countries with good disease surveillance system and applies a natural history model for countries with poorer disease control program and surveillance system, and the latter is a cohort model incorporating country-specific cause-of-death (CoD) profiles among children under-five. The trends of estimates of the two models are similar, but the estimates of the first year are different in most of the countries included in the analysis. The two models are comparable if we adjust the measles CoD in the *LiST* to produce the same baseline estimates. In addition, we used the models to estimate the potential impact of stopping using measles vaccine over a 7-year period. The WHO/IVB model produced similar estimates to the *LiST* model with adjusted CoD. But the *LiST* model produced low estimates for countries with very low or eliminated measles infection that may be inappropriate.

**Conclusions:**

The study presents methodological and quantitative comparisons between the WHO/IVB and the *LiST* measles models that highlights differences in model structures and may help users to better interpret and contrast estimates of the measles death from the two models. The major differences are resulted from the usage of case-fatality rate (CFR) in the WHO/IVB model and the CoD profile in the *LiST*. Both models have their own advantages and limitations. Users should be aware of the issue and apply as update country parameters as possible. Advanced models are expected to validate the policy-planning tools in the future.

## Introduction

The Lives Saved Tool (*LiST*) was developed to estimate the impact of proven interventions on maternal, child and neonatal mortality. Unlike many existing models, this tool was designed to estimate the impact of multiple interventions simultaneously. One can estimate and compare the impact of intervention options, for example using resources to scale up birth delivery services versus increasing coverage of interventions that could be delivered by community workers. As is outlined in other papers in this volume and in previous publications [1-3], *LiST* is increasingly being used by donor organizations and national programs as part of the overall planning process for maternal, neonatal and child health, including estimates of the impact of measles vaccine on under-five mortality.

WHO produces estimates of annual measles burden, i.e. number of cases, death and disability-adjusted life years (DALYs), which serves as a routine source of global measles burden estimates to monitor progress of measles control [4,5]. Details of the methodology of the estimates, constructed by experts in World Health Organization's Department of Immunization, Vaccines and Biologicals (WHO/IVB), was published in 2007 along with estimates of the trends in deaths due to measles over the period 1999-2005 [5]. These estimates along with the yearly updates on estimates deaths in children under-age five due to measles [5,6] are a major indicator tracking progress towards the goal of measles elimination.

In this paper, we compare and contrast the methods, assumptions and outputs of the *LiST* and the WHO/IVB models as they relate to estimating the impact of measles vaccine on under-five mortality. The paper provides a brief description of the approach used in the two models [5,7,8], a comparison of the assumptions and their sources for the two models and a comparison of the estimates of deaths and the temporal trends from the two model. The paper concludes with a discussion of the relative differences between the models and their strengths and weaknesses.

## Model description and method comparison

In this section, we summarize the WHO/IVB model and *LiST* model, and contrast the key properties and components in the two models.

### WHO/IVB measles model

**Figure 1:**
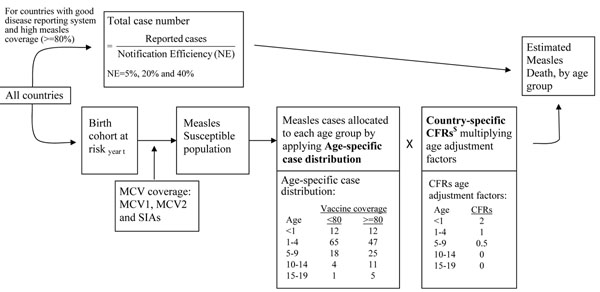
**Diagrams of the WHO/IVB measles model and the *LiST* measles model.** $ Country-specific CFR ranges from 0.05% in the developed countries to 6-8% in the least developed countries

The WHO/IVB model is a mixed model used to estimate country measles burden and to monitor the progress of measles elimination [5]. To best reflect the reliability of country disease surveillance system, countries are first divided into 2 groups according to their quality of disease reporting system (based on experts’ judgment) and annual coverage of routine measles-containing vaccine (MCV) (Figure 1). Different approaches of measles burden estimation are applied to each country grouping. First, countries having high average MCV coverage (>80%) and good disease reporting systems are categorized as group 1. Measles cases are derived from country reports with adjustments of notification efficiency (5%, 20%, and 40%) to accommodate the quality of reporting system. The lower value indicates a better reporting system. Group 2 countries are countries that have poorer disease reporting system or lower MCV coverage that indicates a poorer disease control program. For these countries a natural history model is applied to obtain number of measles cases and death by estimating the proportion of population not protected by MCV immunization under 19 years old [5].

In this paper, we focused on the natural history model estimating measles death for countries with poorer reporting systems. The natural history model first identifies the proportion of population aged 6 month to 19 years old susceptible to measles infection and assumes that all susceptible subjects would acquire measles infection by 20^th^ birthday (Figure 1, details of the model is available in Additional File 1, Appendix 1). The measles susceptible population denotes people who do not receive MCV or who do not develop proper immunity against measles after vaccination. The WHO/IVB model can include probable measles outbreaks when country-reported measles case number is higher than estimated case number. After identifying the size of susceptible population (number of cases), the model allocates cases into 5 age groups using measles case age-specific distributions. Seventy-seven percent measles cases occur to children aged 6 month to 5 years old.

Measles deaths are produced by the multiplication of the number of cases in each age group and age-specific case fatality ratios (CFRs). The WHO/IVB model is illustrated as:(1)

Measles immunization includes two routine MCV (MCV1, administered at 9-12 month old and MCV2, usually administered before children entering primary school) and supplementary immunization activities (SIA) which children receive independently. SIAs in the target year and the previous 4 years are incorporated to account for their effect of reducing measles cases with a series of adjustment factors (100%, 90%, 80%, 50%, and 25%). The model applies a vaccine effectiveness (VE) of 85% for children receiving 1 dose at age younger than 1 year old, and 95% for children receiving more than 1 dose or receiving vaccines after 1 year old.

One major assumption of the WHO/IVB model is that the susceptible population of each birth cohort would acquire measles infection and become cases before their 20^th^ birthday, and a proportion of cases, which is indicated by age-specific CFR, would die from measles. That is to say, the model assumes children surviving through 6 month old do not die of other causes, e.g. diarrhea, non-measles pneumonia, and etc, and will get measles infection before their 20^th^ birthday. This assumption is quite strong since it does not account for the fact that competing diseases cause more death in children under 5 years old than measles infection does. In two reviews of estimating global disease burden in children under 5 years old in 2000-2003 and 2008 [9,10], measles infection accounted for only 6.35% and 1.69% of all-cause under 5 mortality (not including neonatal mortality). It is more appropriate to include the competing causes of death. In addition, infants younger than 6 month old are not included since most of them are protected by maternal antibody against measles infection [11,12]. The distribution of age of infection is derived from epidemiologic studies which suggest 77% of the susceptible children get measles infection before their 5^th^ birthday in a country with low and moderate MCV coverage [13,14]. Because of the high transmissibility of measles, incidence of measles infection among young children decreases only when vaccine coverage is high. The age of infection is shifted to older ages when MCV coverage is higher than 80% with the proportion of children having their measles infection before 5 years old decreases from 77% to 59%.

Country- and age-specific CFR is another key parameter which indicates the proportion of measles deaths among measles cases for each age group. The values of country-specific CFRs are derived from national data or experts’ judge, and are not updated from time to time. However, CFR varies year by year and across graphical areas. It is worthy highlighting the impact of the CFR value on the estimate of measles deaths, e.g. applying a 4% CFR to a given country which has true CFR as 5% produces a measles death estimate as 80% (4%/5%) of the true measles death estimates. The model is sensitive to the variation of CFR. Given the high variation and uncertainty of country-specific CFR estimates [15], it is important to make effort on obtaining the accurate CFR value. Moreover, the model assumes CFR in infants is twice that in children aged 1-4 years old, and is four times of that in children aged 5-9 years old. CFR is 0 for children aged 9 years and older. The assumption is derived by experts’ judge [5,13].

As the authors mentioned in the original publication of the model, the WHO/IVB model is not perfect, but its straightforwardness and transparency can help health authorities understand the epidemics in their country and evaluate their own measles control program. The model is not designed to predict the measles burden for country planning. On the contrary, it aims to understand the current status of measles infection by using available reported and coverage data. The model considers measles infection as the only cause of death and measles vaccine as the only intervention; competing causes of death and other interventions are not considered.

### Measles in the *LiST* software

*LiST* software is a planning tool allowing users to project the concurrent impact of maternal, neonatal and child health interventions, and helps policy-makers to better allocate the limited resources. *LiST* is a cohort model built in the Spectrum Policy Modeling System [7]. The model applies mortality and health indicators to demographic projections (2008 UN Population Division), and models the impact of coverage changes of intervention on population health outcomes. Country-specific cause-of-death (CoD) profile indicates proportions of death caused by measles, diarrhea, pneumonia, and several neonatal causes among neonates and child (aged 1 to 59 month) deaths. The profile was constructed by the WHO Child Health Epidemiology Reference Group (CHERG) based on reviews of peer-reviewed articles, international investigations, national reports and local reports [8,10,16]. The CoD profile is available for year 2000-2003 and 2008 [9,10] and is one of the most important components of the *LiST* module. Detailed method of *LiST* estimates were described in the previous publications [7,8].

**Figure 2:**
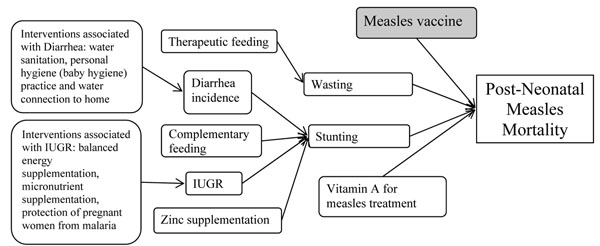
Health status and interventions related to post-neonatal measles mortality in the *LiST*

Measles infection was one of the causes of death in the *LiST* software. Four factors, including two health outcomes (stunting and wasting) and two interventions (MCV coverage and the availability of curative vitamin A supplement in the population), are associated with measles death (Figure 2). The model considers the concurrent coverage and prevalence changes of the four factors and estimates the measles death averted with coverage change of the factors.

**Figure 3:**
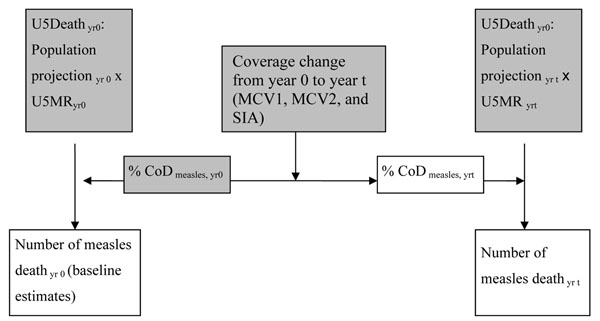
**Diagram of *LiST* measles model.** Grey blocks:input/known parameters White blocks: output/unknown parameters

In this paper, we consider primarily the impact of MCV immunization on measles deaths in the *LiST* model. The *LiST* measles model includes two major parts: baseline estimate and temporal estimates over time (Figure 3). The baseline estimate of under-five measles death is determined by the product of measles CoD proportion, under-five mortality and demographic projection, and is not affected by the baseline MCV coverage. The accuracy of the measles CoD proportion is crucial to the measles death estimates in the *LiST*. Similar to the CFR in the WHO/IVB model, identify measles death among all under-five mortality as 4% instead of the true value 5% would produce the measles death estimates which is 80% of the accurate estimates. Measles death in the target year (yr t) is determined by the change of proportion of population protected which is estimated using MCV coverage change from baseline year and year t and vaccine effectiveness (VE). In other words, by the difference between proportions of children population immune to measles infection by measles vaccination, see the following simplified formula:

The estimates of the baseline year (year 0):(2)

the estimates of the target year (year t):(3)

(Here MCV_yr t_ indicates the coverage of MCV in year t. Mortality rate indicates the mortality for children aged 6 months to 5 years old.)

The *LiST* model incorporated three MCV coverages including the coverage of first and second routine dose and coverage of supplementary immunization activity in each year. Overall MCV coverage is produced by combining the three coverages for each year using the formula presented in Additional File 1, Appendix 2. The impact of SIAs can be traced back to four years prior to the target year [17] but the remaining impact has not been quantitatively identified. We assume a series of SIA weighting factor (100%, 88%, 64.7%, 41.2%, and 17.6%) to identify the proportion of children aged 6 month to 5 years old in the target year who receive the supplementary dose in the target year or anytime in the previous 4 years. In addition, we assume that the receipt of routine MCV doses is independent from receipt of SIAs while the receipt of second routine dose is dependent on receipt of the first one. Different VEs are applied corresponding to the number of doses received and age of vaccine receipt that 85% is for 1 routine dose, 94.2% is for receiving SIA only, and 98% is for receiving 2 doses [18]. Details are presented in Additional File 1, Appendix 2.

Another significant characteristic in the *LiST* measles model is the ability to incorporate the herd effect of measles immunization. The herd effect parameter in the *LiST* indicates the proportion of measles susceptible population could be protected by the indirect effect of the intervention. Based on the result of a systematic review [18], we assume measles infection is blocked when 95% of the population is immune to measles infection by vaccination. The *LiST* software assumes that the herd effect of MCV takes effect when 90% of the population are protected by MCV and achieves total interruption of transmission when 95% of the population immune to measles.

In summary, the *LiST* measles model captures population-specific measles burden by applying an assumed country-specific cause of death structure which provides information on the interaction between causes of death and can incorporate the contribution of multiple interventions associated with measles death.

### Methodology comparison of the two models

As previously explained, WHO/IVB model and the *LiST* model differ from their principal approaches of estimating measles deaths. Table 1 showed parameter-by-parameter comparison between the two models. In the following section, we compare the two models by estimating the first year (baseline value) and temporal trend of estimates of measles deaths, in order to illustrate the differences between the two models.

The baseline value indicates the estimates of measles death for the first year of the targeted time-period. In WHO/IVB model, the estimates are highly dependent on the CFR and MCV coverage (E. 1), both of which vary across countries and over time. In the *LiST*, the CoD profile is the key parameter which incorporates the effect of competing cause of death in the given country (E. 2). To sum up, the difference between the estimates of the first year of the two models depends on how different the estimates derived from natural history model and the estimated obtained by using CHERG review on empirical data on child mortality.

**Table 1:** Comparison of two models in terms of characteristics and parameters used

	WHO/IVB	*LiST*
Model	Natural history model	Cohort model
Population	6month to 59 months, UN Population Division projection, revision 2008	6month to 59 months, UN Population Division projection, revision 2008
Coverage of measles vaccination	MCV1, MCV2 and SIAs [WHO/UNICEF vaccine coverage estimates]	MCV1, MCV2 and SIAs [WHO/UNICEF vaccine coverage estimates]
Protective effect of SIAs	Account for SIAs in the target and the past 4 years Weighting factor: 100%, 90%, 80%, 50%, and 25%	Account for SIAs in the target and the past 4 years Weighting factor: 100%, 88%, 64.7%, 41.2%, and 17.6%
Assumption of independence on vaccine receipt	Assume children receive all three doses independently	Assume children receive the second routine dose on top of receiving the first one. Assume receiving routine doses is independent from receiving SIAs
Coverage being able to distinguish for different age group	Model could be modified to account for that if country-specific information is available	Yes, if country-specific information is available
U5MR	Not applicable	LiST model uses it for computing number of death [UN estimates]
Vaccine Effectiveness	85% for dose 1, 95% for dose 2 and SIAs	85% for receiving MCV1 only, 94.2% for receiving SIA only and 98% for receiving more than 1 doses
Herd effect	Not applicable	Herd effect (HE) kicked in when proportion of population protected by MCV reached 90%. Twenty percent HE was added with 1% increment of protection proportion over 90%. HE reached 100% at 95% of the population directly protected by the vaccine, and all children who do not receive MCV would be protected.
Considering other cause of death	Not applicable	Yes Country-specific cause-of-death profile
Considering other intervention associated with measles infection or mortality	Not applicable	Yes Stunting, wasting, and vitamin A supplement
Country-specific CFRs / CFRs multiplication factor	Yes Age-specific CFR, but the value could not adjust the value with availability of treatments or other intervention	Not applicable
Age-specific case distribution	Yes Different age distribution of cases owing to measles coverage higher or lower than 80%	Not applicable

The temporal trend of measles death is mainly driven by the MCV coverage change over time in both models. The ratios of the measles death of year t to the measles death of the first year are the similar in both models, which are equal to the ratio of the susceptible population of year t to that of the baseline year. The trend differences between the two models are resulted from the calculation of the proportion of susceptible population which indicates the population receiving MCV1, MCV2 and SIAs, and the VEs applied. Different discounting factors of SIAs and assumption of receiving two routine doses independently may cause at most 15% differences of the proportion of population immune to measles infection (Table 1). Furthermore, the *LiST* model incorporates the herd effect of MCV, but the WHO/IVB model does not. The WHO/IVB model incorporates potential country outbreak, but the *LiST* model does not. However, neither model could predict the prospective occurrence or the impact of future measles outbreak.

Considering both the characteristics and differences of the baseline estimates and temporal trend between two models, the two models mainly differ between the estimates of the first year (baseline year) with similar temporal trend. Once we adjust baseline to make similar estimates of the first year in both models by adjusting measles CoD profile in the *LiST* or CFR in the WHO/IVB model, we expect to obtain similar estimate series in both models.

## Numerical comparison of estimates based on historical data between two models

In this section we conducted numerical comparisons to understand the similarity or differences between the two models among children under 5 years old in 2000 to 2007. We adjusted parameters to study how different parameter values affected the estimates in each model and identified potential determinants associated with the differences.

### Data source

We used historical estimates including MCV coverage, mortality, CFR, and CoD profile to perform estimates of both models. We applied population projection from UN Population Division (revision 2008) in both models. The WHO Department of Immunization, Vaccines and Biologicals (IVB) provided the historical MCV coverage of first routine dose (MCV1), second routine dose (MCV2) and supplementary immunization activity (SIA) for 1980-2007. The historical coverage will be publicly released in the future [19-21]. They also provided country-specific CFR used in the original WHO estimates [5] that varied from 0.05% in the developed areas to 8% in the least developed area. Mortality data, including NNMR and U5MR used in the *LiST* model were from *The state of the world’s children*, *2000*[22]. In the *LiST* model, we applied country-specific CoD profile for the baseline year (year 2000) [10,23]. Proportion of measles death ranged from <0.1% (Latin American countries, South Africa, Botswana, and etc) to 8% (Senegal).

### Analysis

First, we applied country-specific MCV coverage, mortality, CoD profile and CFR data in the two models to estimate under-5 measles death between 2000 and 2007. Sixty-eight *LiST*-included countries from 6 WHO regions were included in the analysis (Additional File 1, Appendix 3). Coverage for interventions other than MCV remained constant. Second, we adjusted country specific CoD profiles in the *LiST* to produce same baseline measles death to WHO/IVB estimates*.* We adjusted measles CoD by multiplying the original measles CoD with the ratio of WHO/IVB estimates to the *LiST* estimates for the first year:

We obtained the uncertainty bounds of the WHO/IVB model following the method presented in the original study of the WHO/IVB model in that the researchers applied a 5% absolute higher or lower value of the routine MCV coverage and 20% relative higher or lower of the original CFR in the WHO/IVB model [5]. We produced country-specific estimates for 68 countries in the two models and grouped the countries to present global and 6 regional estimates according to WHO region categories [24], respectively. Here we should keep in mind that uncertainty bounds of the WHO/IVB model were calculated considering variation of the coverage of first routine dose and the CFRs, but not incorporating the variation of the coverage of second routine dose and SIAs. Uncertainty bounds could be narrow with low coverage of MCV1 and high coverages of recent SIAs. However, the method has been used as part of the routine source of measles death estimates, and is familiar to the public health community. So we keep the method and believe it could facilitate communication and understanding in the model comparison.

In addition, we examined the performance of the two models when overall proportion of population protected by MCV decreased from the historical value in 2000 to a presumable 0% in 2007. The proportion of protected population between 2000 and 2007 were the interpolation between the value in 2000 and 0. We performed the measles death estimates for the 68 *LiST* countries, 6 WHO regions and global level, respectively.

## Results

### Estimates of measles death based on historical coverage data in the two models

**Figure 4:**
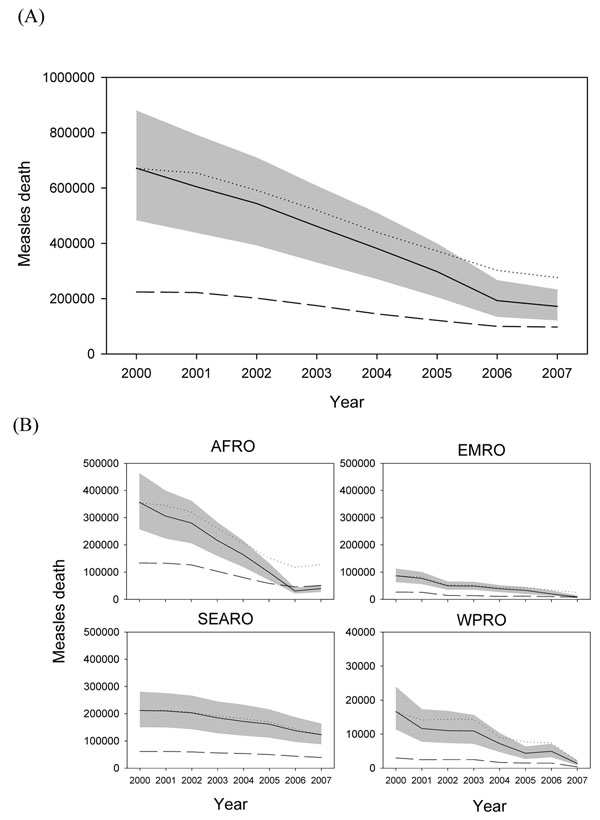
Comparison of the WHO/IVB estimates and the *LiST* estimates, 2000-2007 **(A) Estimates of measles death in 68 *LiST* countries, 2000-2007.** Solid line: WHO/IVB estimates Dashed line: *LiST* estimates Dotted line: CoD adjusted *LiST* estimates Grey area: uncertainty bounds of WHO/IVB estimates. **(B) Regional estimates of measles death in 2000-2007.^#^** # Please note the scale of measles death is different for WPRO. Solid line: WHO/IVB estimates Dashed line: *LiST* estimates Dotted line: CoD adjusted *LiST* estimates Grey area: uncertainty bounds of WHO/IVB estimates.

The *LiST* model and WHO/IVB estimates using historical MCV coverage produced estimates with similar trend in most of the 68 countries in 2000-2007. However, the estimates were very different at the baseline. At the global level, WHO/IVB model estimated a total 671,521 measles (uncertainty bounds: 485,248 to 878,341; Figure 4A) death of the 68 countries in 2000 which was higher than the *LiST* estimates (224,084 in 2000), and the differences between the two models decreased over time. The estimates of the two models were much more similar in 2006 and 2007 at the time coverage had largely increased all over the world. The updated CHERG review on the under-five mortality estimated around 118,000 annual global measles death in 193 countries in 2008 [9] which is between the *LiST* estimates (97,097) of 2007 and the estimate (172,044) in the WHO/IVB model. The updated CHERG review can serve as an external reference based on the epidemiologic studies and country studies. Second, we adjusted the country-specific CoD profile. The *LiST* model produced estimates similar to the WHO/IVB estimates and fell within the uncertainty bounds of the WHO/IVB estimates in 2000-2005.

The performance of the two models revealed regional differences. The estimates were qualitatively similar in both models in other WHO regions, but unadjusted *LiST* estimates were lower than WHO/IVB estimates in AFRO, EMRO, SEARO and WPRO (Figure 4B). In AMRO and EURO where domestic measles infection was eliminated, the annual measles deaths were lower than 100 and were quite similar in both models with high MCV coverages, low CFRs (<0.05%) or low measles death among all under-five deaths most of which are lower than 0.1% (figures not shown). In advance, we performed CoD-adjusted estimates in *LiST* to study the differences between the two models with reduced baseline difference. The adjusted *LiST* estimates and WHO/IVB series were almost identical for AMRO, SEARO and EURO. In AFRO and EMRO, the estimates were similar for year 2000 to 2005, but the differences increased for 2006 and 2007 which were mainly resulted from the difference of coverage calculation in the two models, including different weighting factor of SIAs, and different VE.

In summary, the original *LiST* estimates were quite different from WHO/IVB estimates at the first year due to the different assumptions about the number of measles deaths in the baseline year of 2000. However, the estimates of measles deaths converged over time, especially 2006-2007, at the time the MCV coverage increased. When we used the same estimates of measles deaths for the baseline year of 2000, the two models produced similar estimates of measles deaths for the whole time period.

Country-specific estimates from the two models were qualitatively similar over time, but were different at the baseline year in general as was observed at global and regional estimates (Figures of 68 countries are presented in Additional File 1, Appendix 4). The CoD-adjusted *LiST* estimate series and the WHO/IVB estimates are alike. However, we observed more discrepancies between the two models at individual country level, e.g. Burkina Faso, Cambodia, Democratic Republic of the Congo, Zambia and etc. Two types of discrepancies were observed. One mismatch occurred when the CoD-adjusted *LiST* estimates and the WHO/IVB estimates were matching well, but slightly fell outside of the uncertainty bound of WHO/IVB estimates when intense SIAs were held in countries, e.g. Afghanistan, Cambodia, Nepal, Papua New Guinea, and etc. The different weighting method of SIA coverages in the past 4 years prior to the target year caused at most 15% difference of the population protected by routine MCV when a 100% covered SIA occurred 2 years prior to the target year. In addition, the method used in the master file of IVB was modified and was slightly different from the method described in the Lancet paper.[5] This intra-method discrepancy added up difference between the two models. On the other hand, countries which had no secondary immunization opportunity, e.g. India, Liberia, Pakistan and etc, had almost identical CoD-adjusted *LiST* estimates and WHO/IVB estimates.

In addition, we found a slight modification of the SIA weighting factor for 2000, the baseline year, in the WHO/IVB model from the method presented in the Lancet paper.[5] Smaller values of weighting factors are used for SIAs held in 1996-1999. Therefore, the change resulted in lower baseline coverages for year 2000 in the WHO/IVB model for countries which held SIAs in 1996 to 1999. The change altered the temporal trend of measles death in the WHO/IVB model. As a result, the trend of CoD-adjusted *LiST* estimates and WHO/IVB estimates were less alike in these countries, including Burkina Faso, Democratic Republic of the Congo, Ethiopia, Guinea-Bissau, Niger, Philippines, South Africa, United Republic of Tanzania, Zambia, and Zimbabwe.

### What would happen if we scale down measles vaccine coverage? -- estimates of presumable coverage change

**Figure 5:**
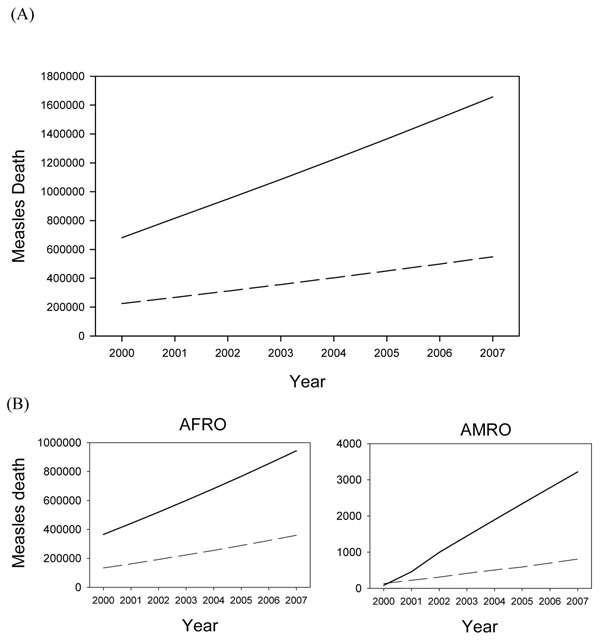
**Total (68 countries) and regional measles death estimates for scaling down MCV coverage from baseline coverage in 2000 to 0% in 2007**. **(A) Total estimates under MCV scaling down scenario.** Solid line: WHO/IVB estimates Dashed line: *LiST* estimates **(B) Regional estimates under MCV scaling down scenario.** Solid line: WHO/IVB estimates Dashed line: *LiST* estimates

We applied the presumable scenario to both models that MCV protecting population went down from the proportion in 2000 to 0% in 2007. Global measles death increased from 680,879 in 2000 to 1,656,922 in 2007 in the WHO/IVB models, while in the *LiST* 224,084 in 2000 to 548,478 in 2007 when no measles vaccine is used in the 68 countries (Figure 5A). The inconsistency of the baseline estimates are still the major difference and the CoD-adjusted *LiST* estimates are quite similar to the WHO/IVB estimate. However, only 805 measles death was predicted for 2007 at the time no measles vaccine was delivered in AMRO where measles had been eliminated in 2000 (Figure 5B).The *LiST* model seemed to produce number of measles death much lower than expected, especially in countries which achieved domestic measles elimination in 2000. Therefore, such few measles death seems inappropriate since measles epidemic will recur quickly after MCV vaccination is scaled down given the high transmissibility of measles virus.

## Conclusions

There are five primary conclusions drawn from the study.

1. The WHO/IVB and the *LiST* model are comparable on measles death estimation from 2000 to 2007, though the baseline value is the major difference of the two models

The temporal trends of measles death of the *LiST* model from 2000 to 2007 are qualitatively similar to the WHO/IVB estimates, though the baseline estimates are significantly different between the two models. When adjusting for the proportion of measles death among all under-five deaths (CoD profile) in the *LiST* model to yield similar baseline estimates to the WHO/IVB model, the two series were comparable. The differences between the CoD-adjusted *LiST* estimates and WHO/IVB estimates were mainly resulted from the different weighting method of SIAs in the two models and also minor modifications of the WHO/IVB method.

2. Cause-of-death profile is the key assumption in the *LiST* model and CFR is critical in the WHO/IVB model

Inconsistency of the baseline values is the major difference between the two models to which parameters, including the CoD-profile in the *LiST* model and the CFR in the WHO/IVB model, are the major determinants. CoD profile was reviewed by a group of experts based on literature, national study and reports [9,10,25]. CFR applied in the WHO/IVB model was review and determined by experts’ review [5]. Both CoD profile and country-specific CFR vary with time and geographical areas. They should be carefully reviewed and updated properly to reflect correct measles epidemic status in each country. Imprecision or inaccurate of the parameters may introduce high uncertainty of the estimates. In addition, the WHO/IVB model considering merely measles infection in the model might have over-estimated the measles death. The results do not suggest that one model is superior or more accurate than another. Readers should be aware of the limitations of the correctness of parameter values and also the assumptions used in either model when interpreting the results of the two models and the comparison between them.

3. *LiST* model does not perform well when measles vaccination is scaled down to very low coverage in a short time period

The model underestimates measles death when scaling down measles vaccination in a short period of time especially in the countries with elimination of domestic measles infection. Owing to the high transmissibility of measles virus, virus could be fast disseminated to the population and cause disease among susceptible population, thus the disease burden, or the proportion of measles death among under-five death, will rapidly increase. Original CoD profile will not serve as a correct parameter under the circumstances. We suggest users to take cautions when they plan to drop the MCV coverage.

4. Multi-intervention associated with measles infection

The study did not pay much attention on the method incorporating multi-intervention related to measles death. The *LiST* model is able to consider the prevalence of stunting, wasting and availability of vitamin A supplement among under-5 children population when estimating measles death while the WHO/IVB model cannot take these factors into account.

5. The decision-making tools are helpful but should be verified by advanced methods in the future

The two models considered in this study are pragmatic and user-friendly which allow country policy planners to plug in and make plan use the country- or population specific data. They demonstrate the convenience and importance though the two models are relatively simple and have their own limitations and uncertainty of estimating under-five measles death. The variation and uncertainty of the key parameters might largely change the results. Therefore, policy-makers who deploy either model to produce country estimates should be aware of the issue and use accurate and updated country-specific parameters for estimation. To accommodate the advantage and disadvantages of these decision-making tools, it is suggested to develop more advanced models, e.g. dynamic transmission models which are able to consider the impact of multiple measles-associated interventions in addition to measles vaccination and to verify the more programmatic tools by comparisons to the advanced models. Similar approach had been taken up in the development and validation of a program-planning tool which estimates the impact of male circumcision on HIV prevention [26].

In this study, we compared the *LiST* model to the widely accepted WHO/IVB model in estimating the impact of measles vaccination on the estimates of measles deaths. There are advantages and disadvantages to both models, but overall the estimates of the impact of scaling up measles vaccine on measles mortality were very similar for the two models. Based on the comparison, we suggested verification of these tools with advanced models should be performed to better understand the validity of the tools. Moreover, it is known that a new WHO measles model is under construction and will be applied by IVB in the near future. Further comparison between the new WHO/IVB model and the *LiST* model should be made when the methodology of the new WHO/IVB model is publically available.

## Competing interests

The authors declare that they have no competing interests.

## Authors' contributions

WC conducted the model comparison, analyses and manuscript preparation.

## Supplementary Material

Additional file 1 Measles model comparison appendix
Appendix 1. Flowchart of WHO natural history model
Appendix 2. Details and assumptions of *LiST* measles model
Appendix 3. Sixty-eight countries included in the analysis, by WHO regions
Appendix 4. Comparison of the WHO/IVB estimates and the *LiST* estimates in 68 countries, 2000-2007^#^ Click here for file
