# Supplementary material for: Comparison of LiST measles mortality model and WHO/IVB measles model
Source: BMC Public Health. 2011 Apr 13;11(Suppl 3):S33. doi: 10.1186/1471-2458-11-S3-S33 (PMC3231907; doi:10.1186/1471-2458-11-S3-S33)
Supplement: Additional file 1 — Measles model comparison appendix Appendix 1. Flowchart of WHO natural history model Appendix 2. Details and assumptions of LiST measles model Appendix 3. Sixty-eight countries included in the analysis, by WHO regions Appendix 4. Comparison of the WHO/IVB estimates and the LiST estimates in 68 countries, 2000-2007# [file 1471-2458-11-S3-S33-S1.doc]

Appendix 1. Flowchart of WHO natural history model

Method 2

Birth cohortyear_x

[UN Population Division, the 2008 revision]^3^

Multiplicative coefficient for CFRs^13^:

AgeCFRs<121-415-90.510-14015-190

Age-specific case distribution^12^: by coverage

Vaccine coverageAge<80>=80<112121-465475-9182510-1441115-1915

Method 1

Case numberyear_x

=

NE=5%, 20% and 40%^2^

For countries with moderate-to-poor measles control^1^

Coverageyear_x (MCV):

dose 1: from WHO/UNICEF national routine coverage^4^

dose 2: country report

SIAs:
 [WHO]^5, 6^

Vaccine effectiveness (VE)^7, 8^:

Age of vaccination < 1 yrs: 85%

Age of vaccination >= 1yrs: 95%

SIAs: 95%

Discounting prospective effect of SIAs^9^:

100, 90, 80, 50, and 25(%) for 0, 1, 2, 3, 4 years after the SIAs

Proportion of Protected ^10^=

1-(1-VE^8^ x MCV1) x (1-VE_>1_ x MCV2) x (1-VE_>1_ x 100% x SIA_i_) x (1-VE_>1_ x 90% x SIA_i-1_) x (1-VE_>1_ x 80% x SIA_i-2_) x (1-VE_>1_ x 50% x SIA_i-3_) x (1-VE_>1_ x 25% x SIA_i-4_)

Cases^11^ = (Births-Deaths in first 6 months of life) x (1-Protected)

X

- Country-specific case fatality rate for children aged 1-4 years.

X

**All countries**

X

For countries with good disease reporting system and high measles coverage (>=80%)^1^

Here we illustrate the details and assumptions used in the WHO/IVB measles model:

1 The determination of using method 1 or 2 was based on the country disease surveillance system and the coverage of measles-containing vaccine (MCV). In brief, countries with good surveillance systems and also routine MCV coverage higher than 80% use method 1. Countries with moderate-to-poor measles control, mostly in Sub-Saharan Africa and South-east Asia, use method 2. The countries applying method 2 may have routine MCV coverage higher than 80% but relatively poor surveillance system or have both low vaccine coverage and poor surveillance system.

2 NE (notification efficient) is determined mainly by expert opinion. However, the estimate of countries by using method 1 doesn’t matter that much, since measles infections and deaths in this group is relatively fewer compared to those in countries using method 2.

3 The birth cohorts are updated with UN Population Division projections. The recent update is 2008 revision.

4 $\text{MCV1}=\frac{\text{number} \text{of} \text{children} \text{received} \text{their} \text{first} \text{of} \text{dose} \text{in} \text{this} \text{year}}{\text{children} \text{reach} \text{their} \text{first} \text{birthday} \text{this} \text{year}}$ (ratio)

5 In most of the cases, countries don’t have both second routine measles vaccination and SIAs at the same time. Besides, the receipt of dose 1 is independent of receipt of supplementary dose.

6 The coverages of SIAs are available in the working file of WHO/IVB

7 Vaccine effectiveness (VE) varies based on the age of receiving the routine MCV and the implementation of recent campaign (in the past 4 years).

8 Measles vaccine effectiveness for first routine dose is modified to 95% if recent campaign (in the past 2 years) was held. Assumptions: The VE in children who received the first routine dose is boosted to 95% if they receive another dose in the SIAs held in 2 years [1].

9 Discounting effect indicate the effect of SIAs. The longer time passes after the campaign, the less effect of reducing measles cases. The effect of reducing measles cases lowers because new susceptible cohorts enter in the population.

10 The method assumes an independent effect of receiving any dose of measles vaccines and the effectiveness is applied

11 This model does not consider competing causes of death.

12 Assuming that all children who are not protected will be infected at some point, and the distribution of their age of infection is age-specific case distribution.

13 The case fatality rates (CFRs) for age groups are estimated based the CFR for children aged 1-4 years multiplied by coefficients assigned to each age group. The coefficients were assigned by experts.

14 The numbers of measles cases in each age group are estimated by the multiplication of age distribution and the overall number of cases. The higher value of either case estimates or reported cases will be used.

# For WHO/IVB model, the determinants of measles death are birth cohort, coverage of routine and campaign. Other components and assumptions in the model are set constant to make the model most standardized and comparable between years and between countries.

Appendix 2. Details and assumptions of LiST measles model

Here we presented details of the methods used in the *LiST* measles model of calculating the proportion of population protected by the measles vaccines.

Table 1 Table illustrating the categories of measles vaccine receipt

|  |  | Routine (MCV1 or MCV1/2) | |  |
| --- | --- | --- | --- | --- |
|  |  | + | - |  |
| SIA | + | A | B | (%SIA) |
|  | - | C | D |  |
|  |  | (% MCV1/2) |  |  |

Table 2. Vaccine effectiveness (VE) and estimated coverage of each category of vaccine receipt

| Category | | Effectiveness^2^ | | |  | | Coverage of each category* |
| --- | --- | --- | --- | --- | --- | --- | --- |
| A | Receive 2 doses or more | | 0.98 |  | | MCV1xMCV2+MCV1x(1-MCV2)xSIA_all_ | |
| B | Receive SIA only | | 0.942 |  | | (1-MCV1)xSIA_all_ | |
| C | Receive first routine dose only | | 0.85 |  | | MCV1x(1-MCV2)x(1-SIA_all_ ) | |

**Input: MCV1, MCV2, SIA (of the target year and of the previous 4 years, respectively)**

**Output: Overall proportion of population protected by MCV**

Overall protection = $\sum\left(\text{VE}\times\text{Coverage}\right)$ = $0.98\times\text{A}+0.942\times\text{B}+0.85\times\text{C}$

The *LiST* software assumes that the herd effect of MCV takes effect when 90% of the population are protected by MCV and achieves total interruption of transmission when 95% of the population immune to measles.

**Assumptions:**

1. Assuming the probability of receiving routine dose and any SIA is independent.
2. Differential VE for children receiving different types (MCV1, MCV2, and SIA) and number of doses of measles [2]
   1. VE for receiving first routine dose (MCV1) : 0.85.
   2. VE of receiving at least 2 doses of measles vaccine: 0.95.
   3. VE for receiving any SIA but not other routine doses: we assumed the VE for receiving any SIA among children aged 9 - 12 months old is 0.85 and the VE for children aged 9-59 months old receiving any SIA is 0.95. Therefore, a combined VE of receiving any SIA among children under-5 years old is 0.942. [$=\frac{\text{12-8}}{\text{59-8}}\times0.85+\frac{\text{59-12}}{\text{59-8}}\times0.95$]
3. Herd effect: In the previous studies, mathematical modeling concluded that newborn measles immunization coverage should exceed 94% to eliminate the transmission [3]. Therefore, we assume herd immunity kicks in overall proportion of population protected by measles vaccines reaches 90% and infection is totally eliminated when 95% of the population is protected by MCV. Models were established with assumption of equivalent coverage across all subgroups in the target population.
4. Vaccine immunogenicity waning: Protection provided by measles vaccination is assumed to last until children’s fifth birthday in the general population. The impact of immunogenicity waning among immuno-deficienty children, e.g. HIV-infected children, were not included in the estimate.
5. Coverage of SIA: supplementary immunization activity (SIA) is held irregular in countries with lower routine coverage or no provision of second routine measles vaccine. In the *LiST* model, we calculated an overall coverage of SIA incorporating the effect of SIA held in the target or in the previous four years using a series of weighting factors.
   1. Weighting factor: Weighting factors are derived according to age-out concept which help us to identify the proportion of children aged 6 month to 59 months old in the target year who receive the supplementary dose in the target year or anytime in the previous 4 years. Children aged 9-59 months old and targeted in a SIA campaign years before the target year might pass the 59 month old threshold in the target year. For example, children who aged 9-59 months old one this year turned into 15-65 months old in the following year. Among them, those who aged 60-65 month old fell out of the target group and would not be included in our target population. Therefore, we generate a series of SIA weighting factor (100%, 88%, 64.7%, 41.2%, and 17.6%) which indicate the proportion of children who are aged 9 to 59 months old in the garget year and receive any SIA in the target or within 4 years prior. The weighting factors are generated as follows:
      - Target year: 100%, previous year: $\text{88\%}=\frac{\text{59-14}}{\text{59-8}}$, two years before: $\text{64.7\%}=\frac{\text{59-26}}{\text{59-8}}$, three years before: $\text{41.2\%}=\frac{\text{59-38}}{\text{59-8}}$, four years before: $\text{17.6\%}=\frac{\text{59-51}}{\text{59-8}}$
   2. Overall SIA coverage for the target year: The estimated coverage of each SIA in a designated year indicates the proportion of children who received SIA and were aged 9-59 month old at mid-year. The overall SIA coverage is calculated as follows:

SIA_all_ = Overall SIA coverage (%) = $100\times\left[\text{1-}\left(\text{1-1}\times\frac{\text{SIA}_{0}}{100}\right)\times\left(\text{1-0.88}\times\frac{\text{SIA}_{\text{-1}}}{100}\right)\times\left(\text{1-0.647}\times\frac{\text{SIA}_{\text{-2}}}{100}\right)\times\left(\text{1-0.412}\times\frac{\text{SIA-3}}{100}\right)\times\left(\text{1-0.176}\times\frac{\text{SIA}_{\text{-4}}}{100}\right)\right]$

References:

1. Wolfson LJ, Strebel PM, Gacic-Dobo M, Hoekstra EJ, McFarland JW, Hersh BS: **Has the 2005 measles mortality reduction goal been achieved? A natural history modelling study**. *Lancet* 2007, **369**(9557):191-200.

2. Sudfeld CR, Navar AM, Halsey NA: **Effectiveness of measles vaccination and vitamin A treatment**. *International journal of epidemiology* 2010, **39 Suppl 1**:i48-55.

3. Hethcote HW: **Measles and rubella in the United States**. *American journal of epidemiology* 1983, **117**(1):2-13.

Appendix 3. Sixty-eight countries included in the analysis, by WHO regions

| Africa (AFRO) | | | [the Americas](http://www.who.int/about/regions/amro/en/index.html)  (AMRO) | [the Eastern Mediterranean](http://www.who.int/about/regions/emro/en/index.html) (EMRO) | Europe (EURO) | [South-East Asia](http://www.who.int/about/regions/searo/en/index.html) (SEAR) | [the Western Pacific](http://www.who.int/about/regions/wpro/en/index.html) (WPRO) |
| --- | --- | --- | --- | --- | --- | --- | --- |
| Angola  Benin  Botswana  Burkina Faso  Burundi  Cameroon  Central African Republic  Chad  Congo  Cote d'Ivoire  Democratic Republic of the Congo  Equatorial Guinea  Eritrea  Ethiopia  Gabon | The Gambia  Ghana  Guinea  Guinea-Bissau  Kenya  Lesotho  Liberia  Madagascar  Malawi  Mali  Mauritania  Mozambique  Niger  Nigeria  Rwanda  Senegal | Sierra Leone  South Africa  Swaziland  United Republic of Tanzania  Togo  Uganda  Zambia  Zimbabwe | Bolivia  Brazil  Guatemala  Haiti  Mexico  Peru | Afghanistan  Djibouti  Egypt  Iraq  Morocco  Pakistan  Somalia  Sudan  Yemen | Azerbaijan  Tajikistan  Turkmenistan | Bangladesh  Democratic People's Republic of Korea  India  Indonesia  Myanmar  Nepal | Cambodia  China  Lao People's Democratic Republic  Papua New Guinea  Philippines |

Appendix 4. Comparison of the WHO/IVB estimates and the *LiST* estimates in 68 countries, 2000-2007^#^


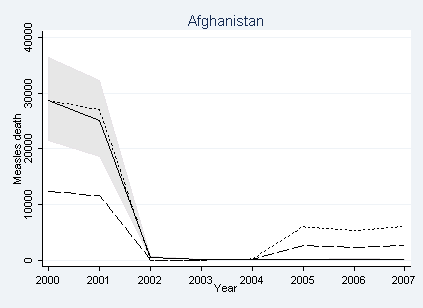

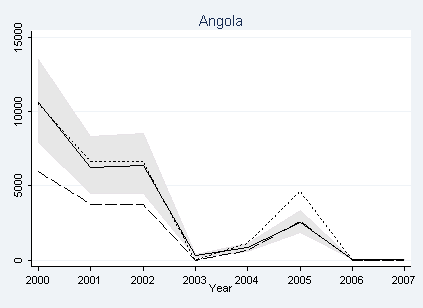

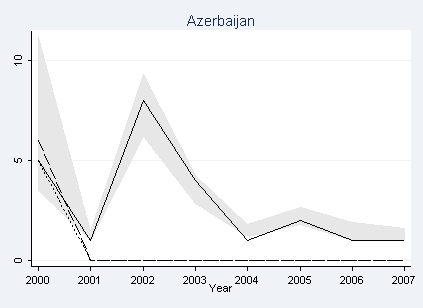

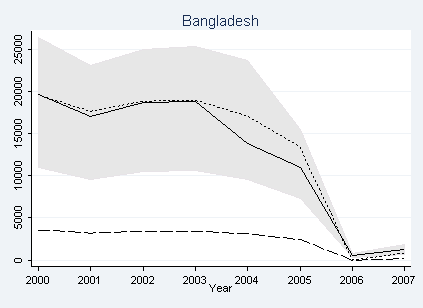

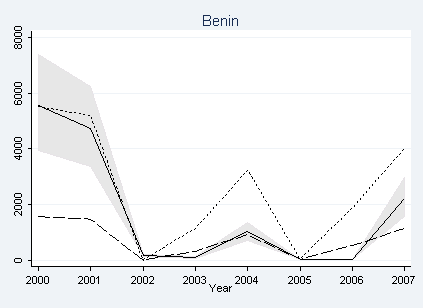

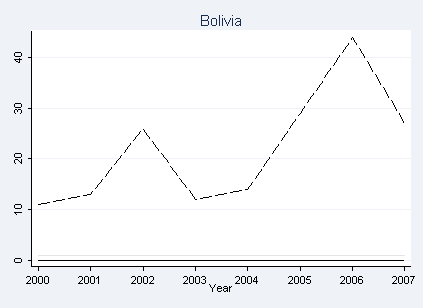

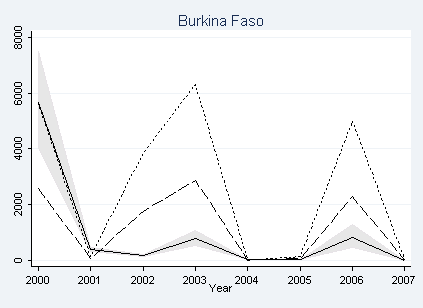

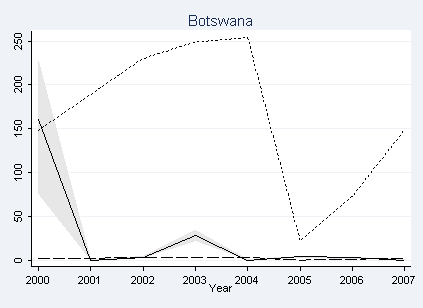

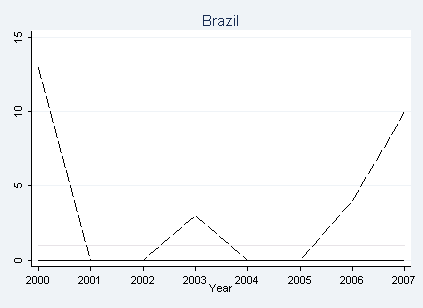

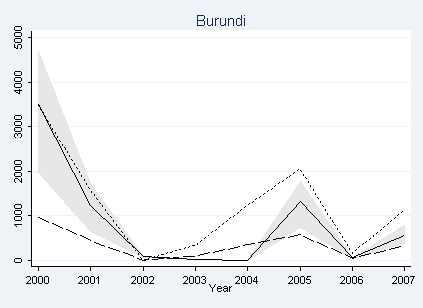

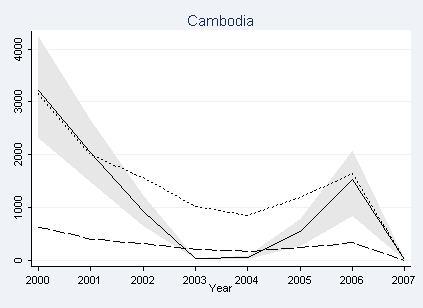

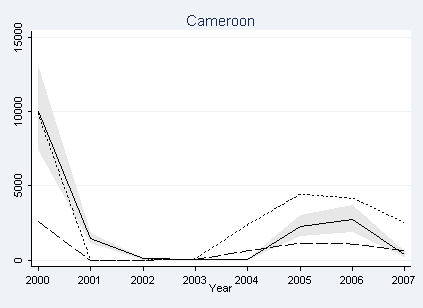

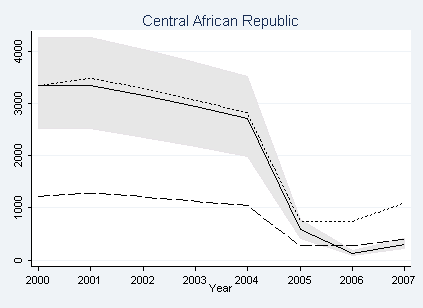

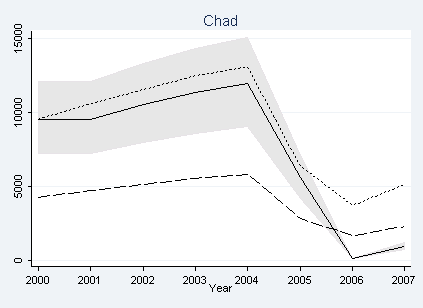

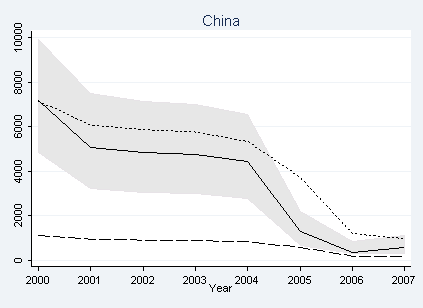

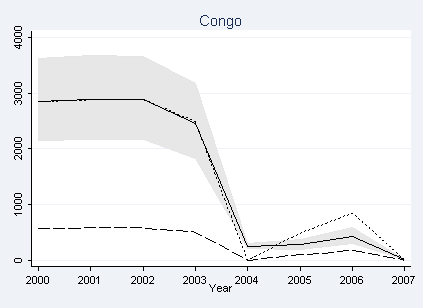

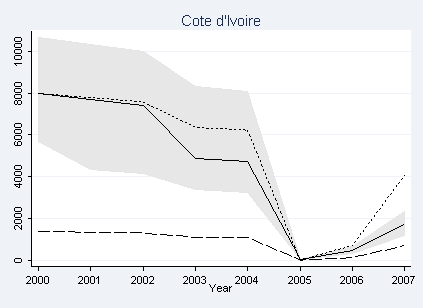

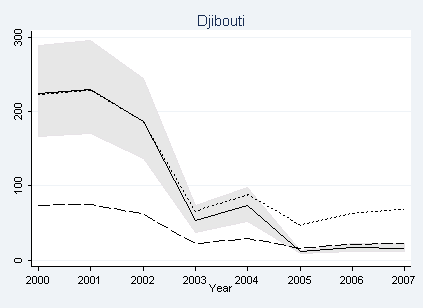

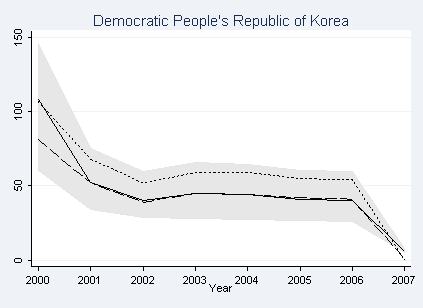

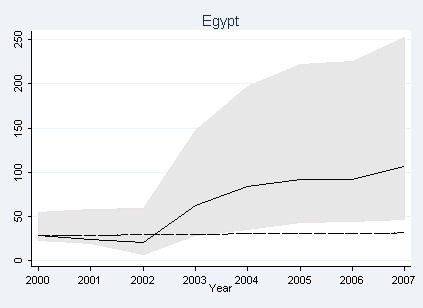

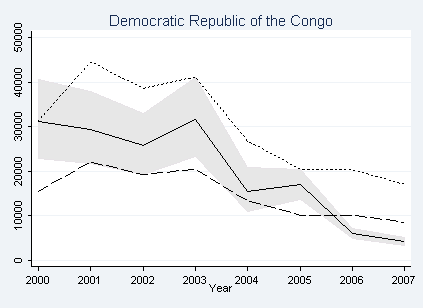

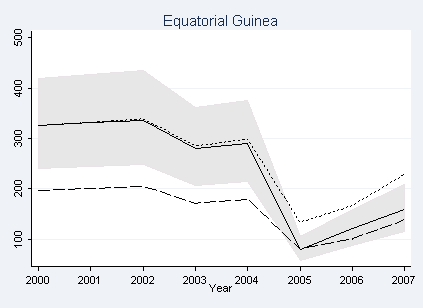

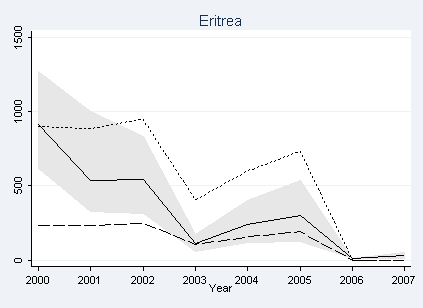

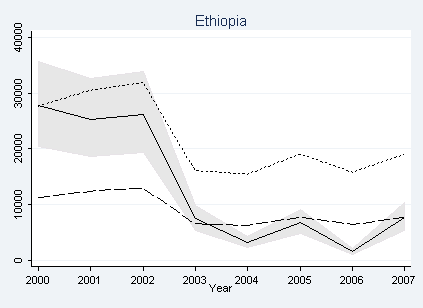

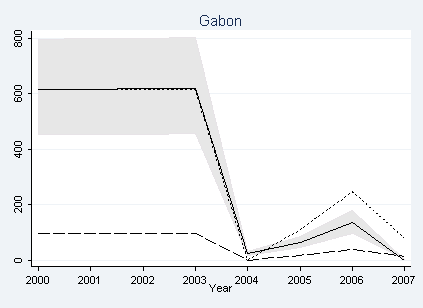

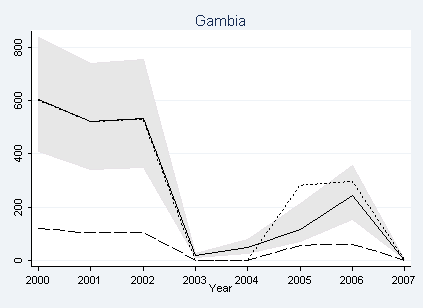

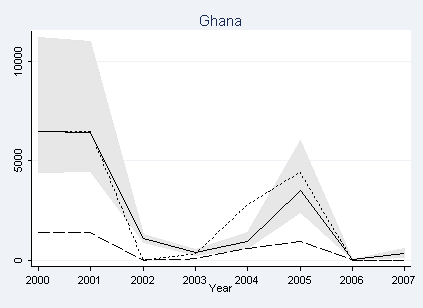

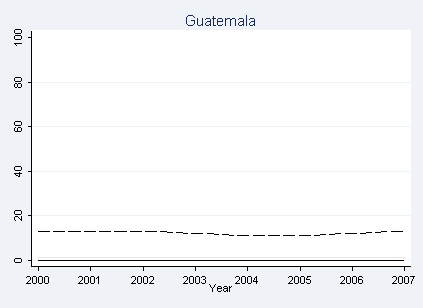

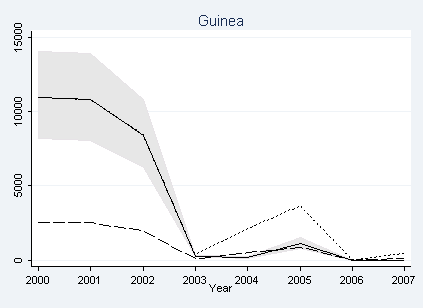

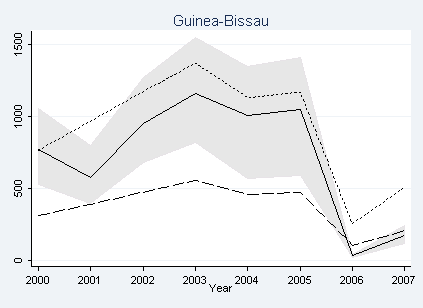

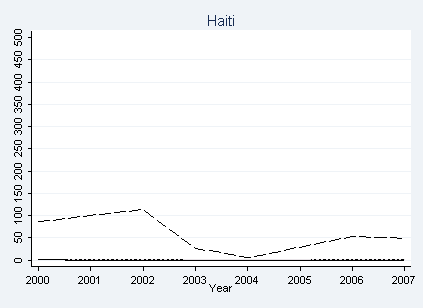

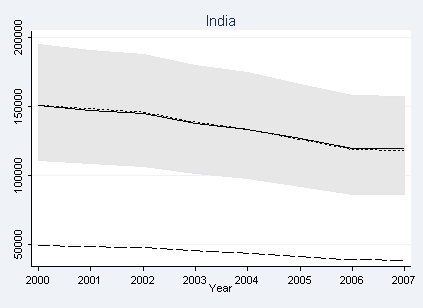

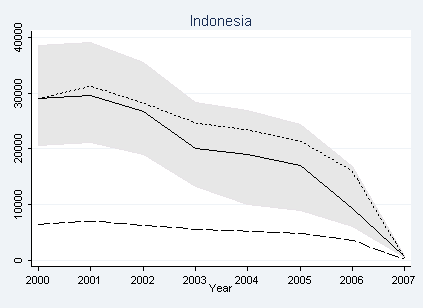

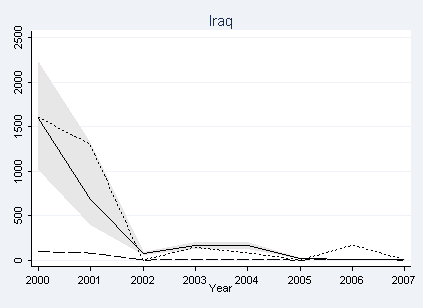

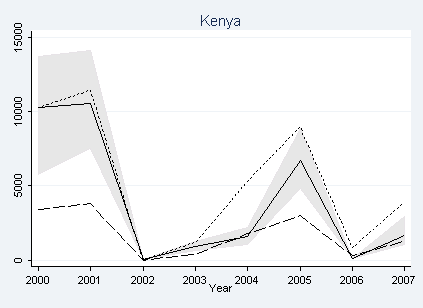

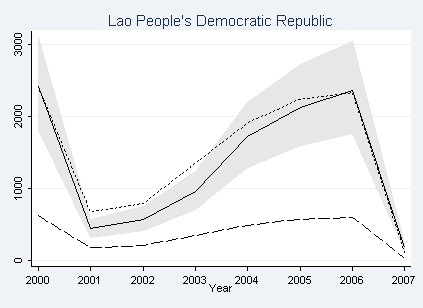

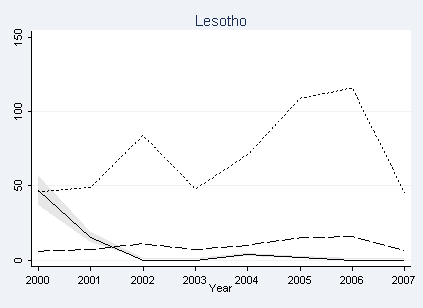

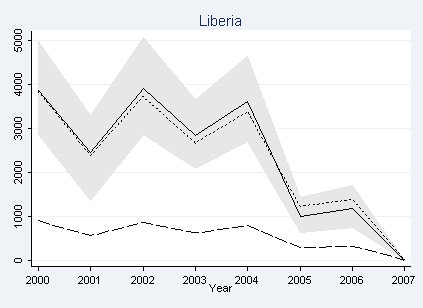

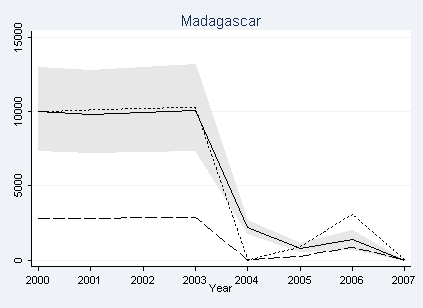

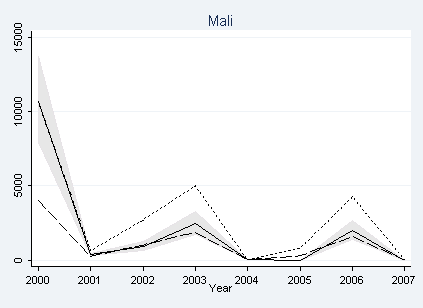

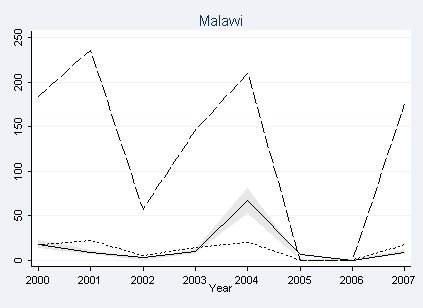

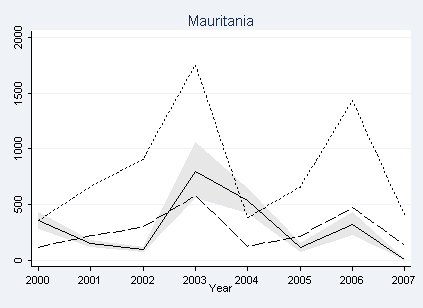

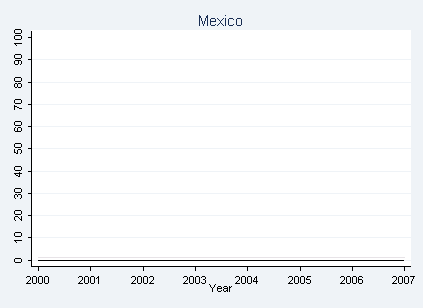

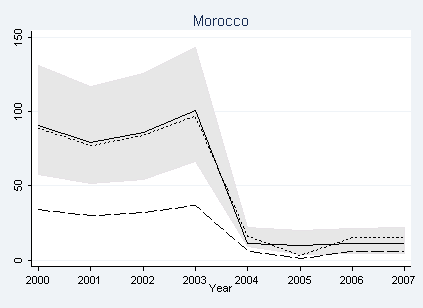

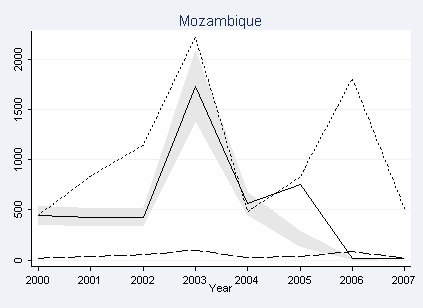

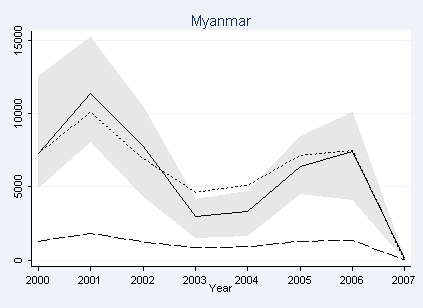

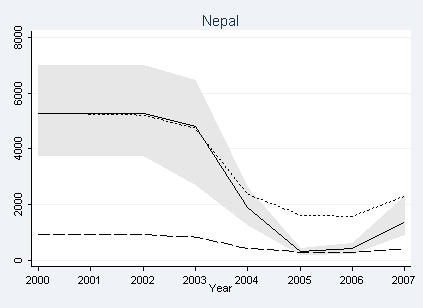

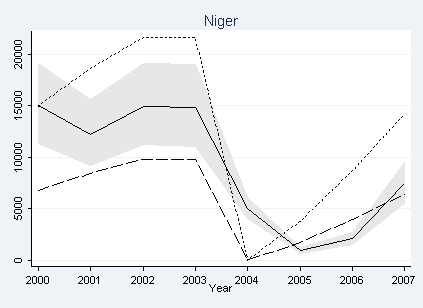

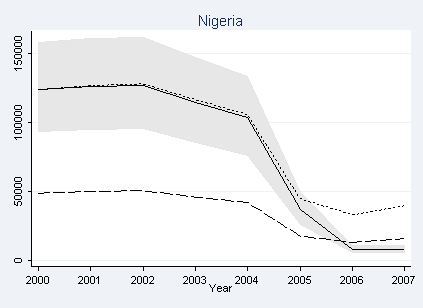

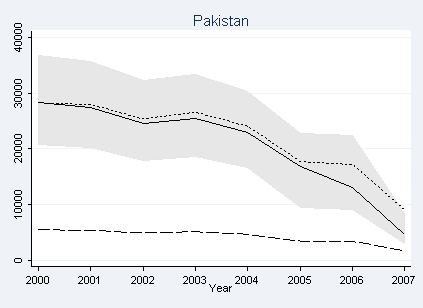

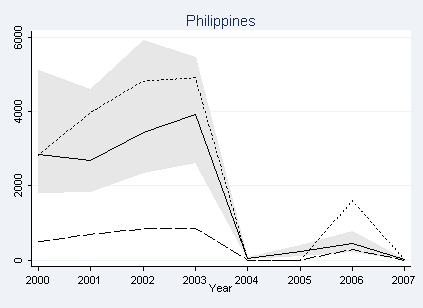

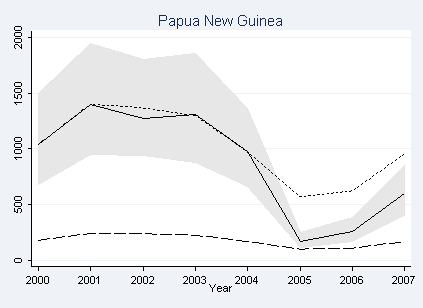

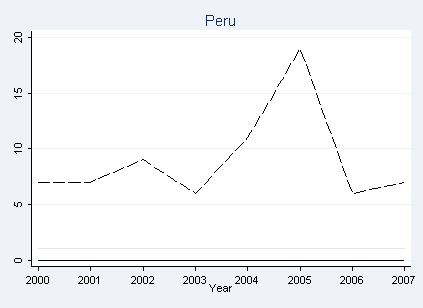

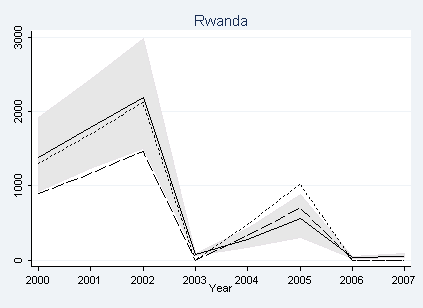

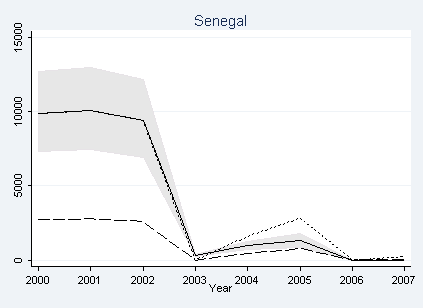

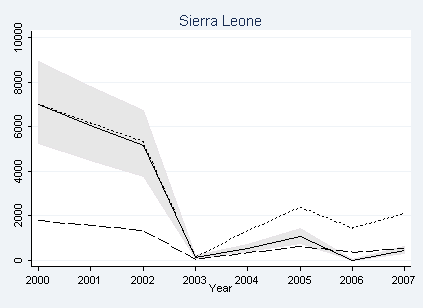

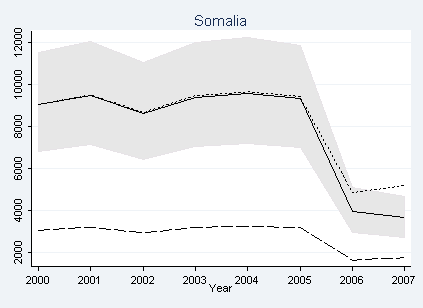

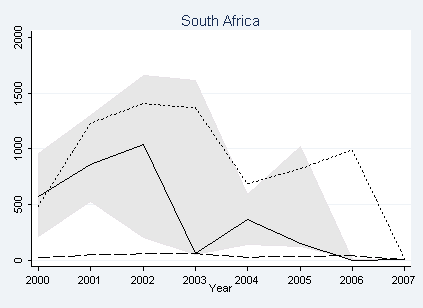

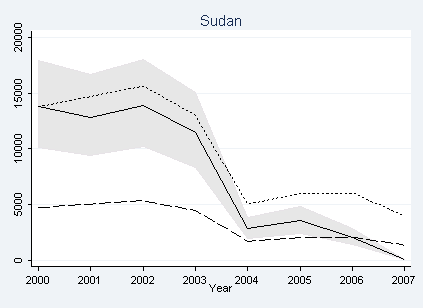

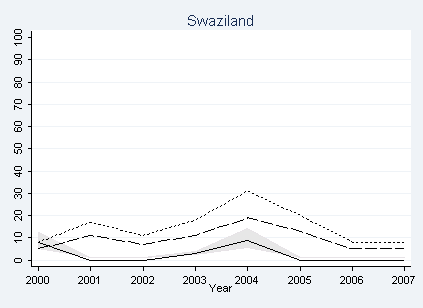

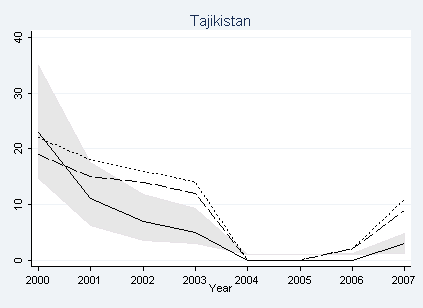

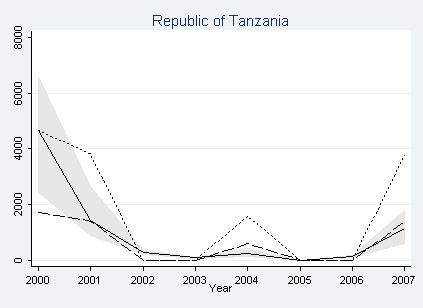

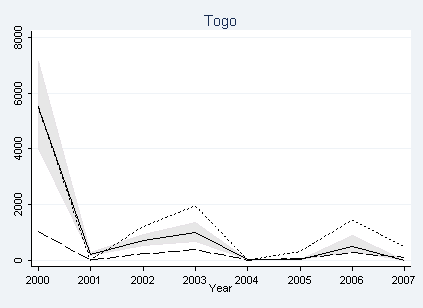

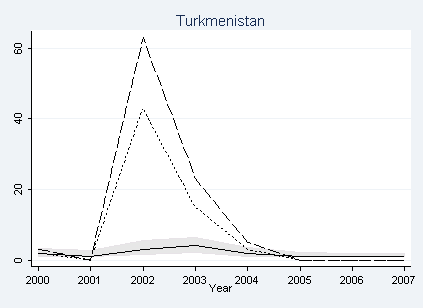

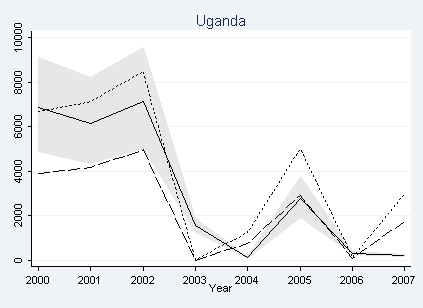

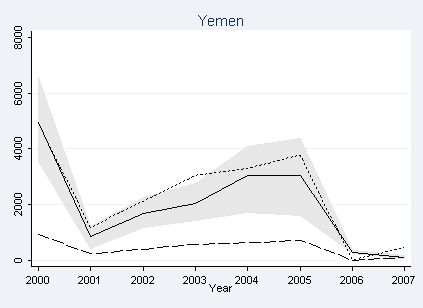

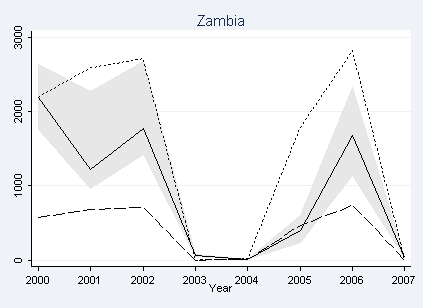

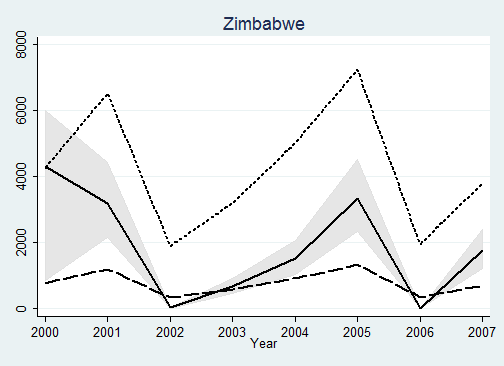


# Note the y scale of measles death might vary over countries.

Y axis indicates numbers of measles death

Solid line: WHO/IVB

Dashed line: LiST

Dotted line: CoD adjusted LiST estimates

Grey area: uncertainty bounds of WHO/IVB estimates
